# *Malalcahuello ocaresi* gen. & sp. n. (Elateridae, Campyloxeninae)

**DOI:** 10.3897/zookeys.508.8926

**Published:** 2015-06-15

**Authors:** Elizabeth T. Arias-Bohart

**Affiliations:** 1 Essig Museum of Entomology, University of California 1101 Valley Life Sciences Building, Berkeley 94720, California, USA

**Keywords:** Coleoptera, Elateridae, Campyloxeninae, *Malalcahuello*, *Campyloxenus*, Chile

## Abstract

*Malalcahuello ocaresi* **gen. n. & sp. n.**, from Chile, is described and compared with *Campyloxenus pyrothorax* Fairmaire & Germain, 1860.

## Introduction

Fairmaire and Germain (1860) described *Campyloxenus pyrothorax*. Costa (1975) transferred the species to his newly erected monotypic subfamily Campyloxeninae based on the following character states: claws lacking setae near base, hind wings with a wedge cell, female genitalia with a stylus and a very elongate baculum. Stibick (1979) placed it within the Agrypninae based on the presence of prothoracic luminous organs. All authors (Golbach 1994; Lawrence et al. 2010a; Bouchard et al. 2011; and Arias-Bohart and Elgueta 2012) have followed Costa (1975) in retaining *Campyloxenus pyrothorax* within its monotypic subfamily Campyloxeninae. During an on-going canopy forest fogging surveys over the last decade in Chile (Arias et al. 2008; Richardson and Arias-Bohart 2011) we collected an unknown click beetle which I describe here and place within the Campyloxeninae.

## Materials and methods

Specimens and primary types repositories are from institutional and private collections. Acronyms follow those provided by the institution or Arnett et al. (1993).

ANIC Australian Insect Collection, Canberra, Australia;

BMNH British Museum of Natural History, London, England;

EMEC Essig Museum of Entomology, University of California, Berkeley, USA;

ETA Elizabeth Arias-Bohart (private collection) Sacramento, USA;

FMNH The Field Museum of Natural History, Chicago, Illinois USA;

MNHN Muséum national d’Histoire naturelle, Paris, France;

MNNC Museo Nacional de Historia Natural, Santiago, Chile;

JEB Juan Enrique Barriga Tuñon, (private collection) Curicó, Chile;

RBINS Collections Nationales Belges d’Insectes et d’Arachnides, Institut royal des Sciences Naturelles de Belgique, Brussels, Belgium;

SRC Sergio Riese (private collection) Genova, Italy.

The following procedure as detailed by Becker (1958) was used for examining male and female genitalia: The last few abdominal segments were removed and placed overnight in in a Petri dish with soapy in order to soften the tissues. Male genitalia were extracted, examined and stored in small genitalia vials with 90% alcohol, or glued to a card pinned under the specimen. Measurements using a calibrated ocular micrometer are as follows: total body length from the frontal margin to elytral apex; pronotal length and maximum width of the pronotum and elytral length and maximum width of elytra. Adult morphology follows Gur’yeva (1974), Platia (1994), Calder (1996), Arias (2008), Lawrence and Arias (2009), Lawrence et al. (2010b), Arias-Bohart (2013, 2014). Wing vein nomenclature follows that of Dolin (1975), Kukalova-Peck and Lawrence (1993, 2004). Locality data were taken directly from labels where / = line separation and // = new label. Approximate GPS, when not available, its provided underlined. Locality data for JEB material can be accessed at http://www.coleoptera-neotropical.org. Drawings were made using a camera lucida on a Leica MZ7 dissecting scope. Drawings were made using a camera lucida on a Leica MZ7 dissecting scope. Type material has been databased with a unique number indicated on the label information consisting of the acronym EMEC and the identification number. For example, the holotype of *Malalcahuello ocaresi* sp. n. has the unique number EMEC117539 that can be accessed at http://essigdb.berkeley.edu.

## Taxonomy

### Malalcahuello gen. n.

Taxon classificationAnimaliaColeopteraElateridae

http://zoobank.org/E87CF381-6C94-49F2-AFB4-832AF2EF264B

#### Type species.

*Malalcahuello ocaresi* sp. n., here designated.

#### Etymology.

The generic name Malalcahuello (gender masculine) is derived from the type locality of origin of the genus, Malalcahuello, in southern Chile. The word Malalcahuello derives from Mapudungun language *malal* = barnyard and *kahuellu* horse (Musigraf 2003).

#### Diagnosis.

This genus differs from all other elaterid genera by the following combination of characters: strongly serrate antennae from antennomere 3 onwards, antennomere 2 very small, length about 0.4 times as long as antennomere 3; pronotum 0.76–0.99 as long as wide, convex, without deep impressions basally, lacking bioluminescent organs; stout, and protruding posterior angles with apex truncate; mesocoxal distance about 0.16 times mesocoxal cavity; wing venation with R cell elongate 4.2 times its width and wedge cell length 4 times its maximum width.

#### Description.

**Figures 1–4.**
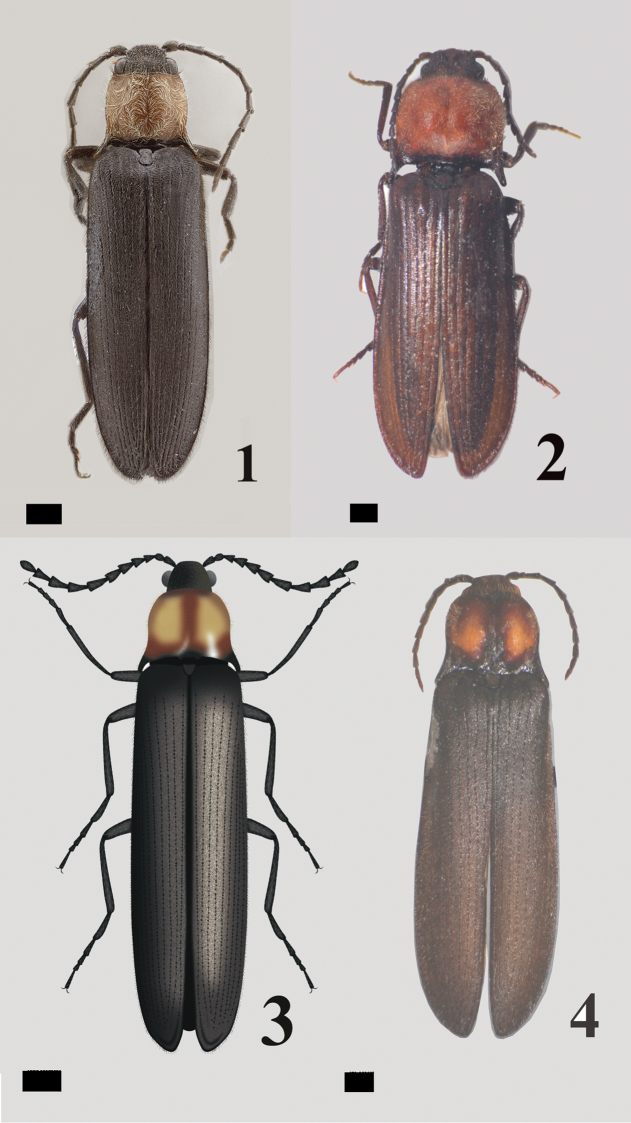
**1–2** Adult *Malalcahuello ocaresi* sp. n.: male (**1**), female (**2**) **3–4** Adult of *Campyloxenus pyrothorax*: male illustration by Nancy Arias Tobar (**3**), female (**4**).

Body about 3.27–3.87 times as long as wide; pronotal sides slightly sinuated, narrower than elytral sides. Elytral maximum width at posterior third; elytral apices softly rounded, not meeting at mid-line. Dorsal vestiture short, spare, fine, with some erect and decumbent short, well distributed hairs (Fig. 1).

**Figures 5–8.**
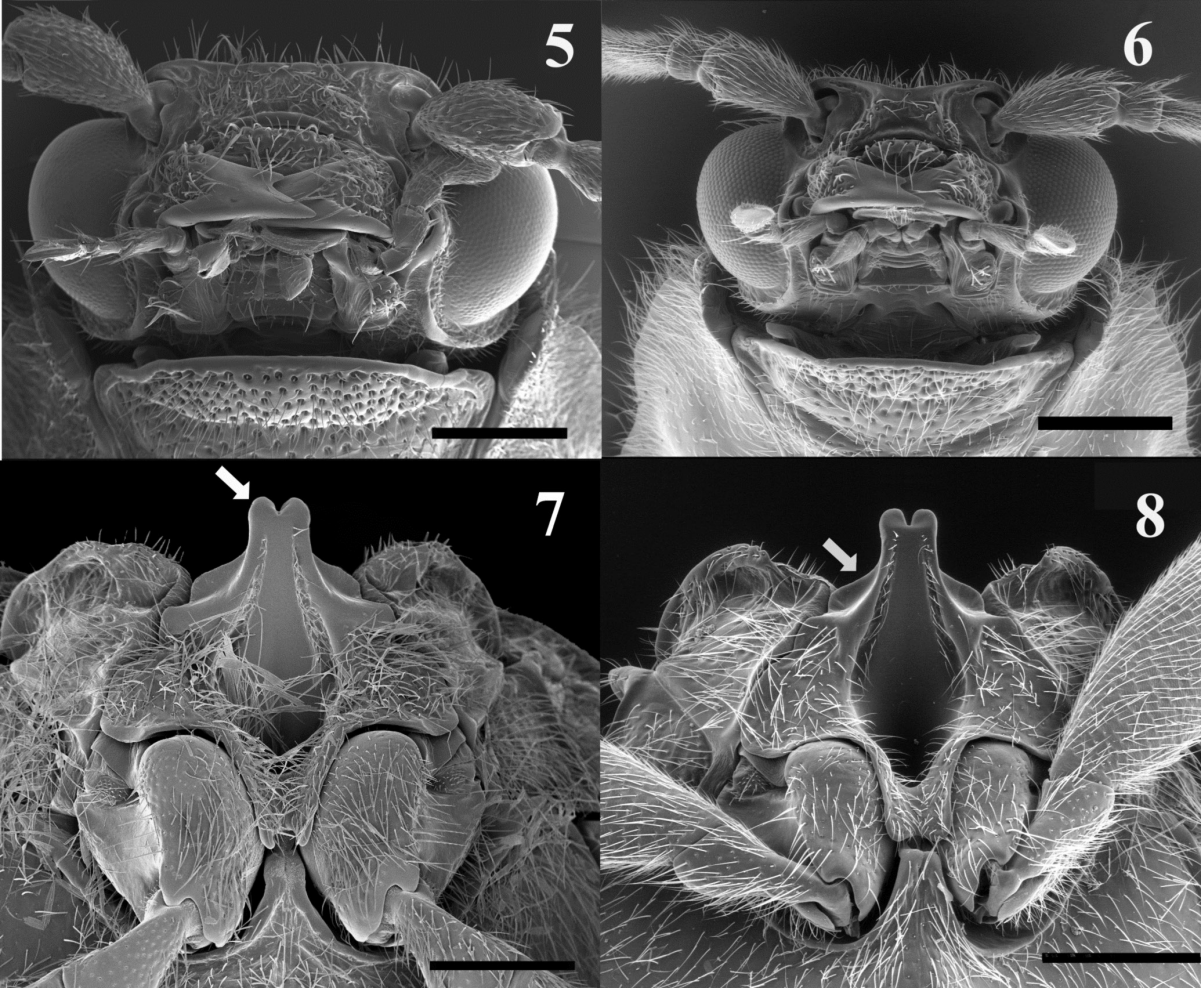
**5–6** SEM illustration of frontal head of: *Malalcahuello ocaresi* (**5**), *Campyloxenus pyrothorax* (**6**) **7–8** SEM illustration of mesoventral cavity of, *Malalcahuello ocaresi* (**7**), *Campyloxenus pyrothorax* (**8**). Scale bar = 0.5 mm.

Head slightly declined at base, transverse, ratio of median length to greatest postocular width 0.30–0.42. Eyes medium size, protuberant in both sexes, facetted, without interfacetal hairs. Supra-antennal ridges raised above, each antennal fossa with deep curved invagination between antennal insertion and eye; short (Fig. 5). Frontoclypeal region completely carinate, produced forward, not concealing clypeus; frontoclypeal carina rugulose; clypeus length about 4.8 times its width. Labrum elongate, sclerotized, sinuate basally.

Antennae in male surpassing posterior angles, antennomeres 3-10 strongly serrate, antennomere 11 elongate, longer than preceding ones; all antennomeres clothed with short, semi-decumbent goldish hairs and few erect, goldish long hairs. Female antennae shorter than male antennae (Fig. 2).

Prothorax subquadrate, sides slightly sinuated, carinate and emarginate, visible for their entire lengths from above; posterior angles stout, protruding, with apex truncate and produced posterolaterally embracing humeral area; posterior edge without scutellar notch; disc finely punctate, clothed with gold sinuated vestiture; prosternum strongly combed, with deep punctures; notosternal suture complete, strongly sinuated, open at anterior end, curved at posterior end; prosternum puncticulate, with semi-erect, sparce hairs; prosternal process slightly narrower near base, then gradually expanded posteriorly, following procoxae in lateral view, extending well behind procoxae. Hypomeron simple, depressed medially, with deep punctures. Procoxae subglobular (Fig. 17).

Scutellum not elevated, flat, anteriorly simple, posteriorly rounded, notched on the sides, all borders well defined, tongue-shaped. Elytra about 2.81–2.54 times as long at midline as greatest width and 4.43–5.02 times as long as pronotum; anterior edge carinate; humeri well developed; parallel-sided at anterior third, gradually enlarging towards posterior third, converging posteriorly, apices rounded, not meeting and central midline. Disc with 10 defined puncture rows.

Mesoventrite on same plane as metaventrite; mesocoxae projecting, mesocoxal cavities narrowly separated, open laterally to mesepisternum; mesocoxal distance 0.25 times mesocoxal diameter; mesosternal posterior region excavated and 0.23 times mesocoxal diameter length (Fig. 7); metacoxae obliquely oriented, with plates extending narrowing towards body side; posterior region of mesosternite length 0.35 times as mesocoxal diameter length.

**Figures 9–16.**
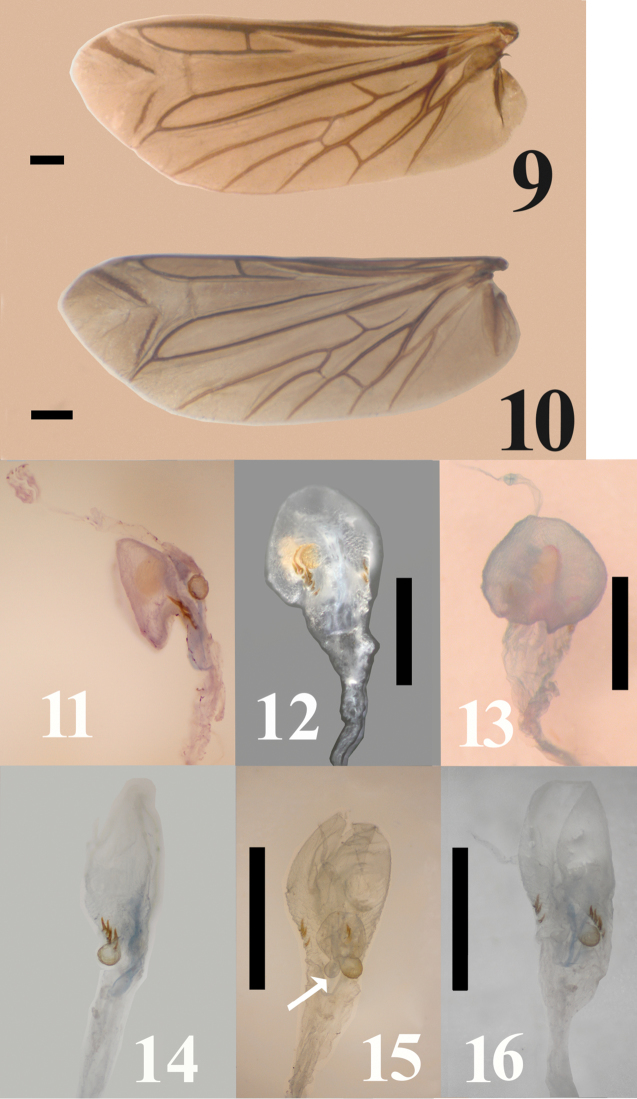
**9–10** Wing venation illustration of *Malalcahuello ocaresi* (**9**), *Campyloxenus pyrothorax* (**10**) **11–13** Female genitalia *Malalcahuello ocaresi* **14–16** Female genitalia *Campyloxenus pyrothorax*. Scale bar = 0.5 mm. (**11–16**); 1 mm (**9–10**).

Hind wing about 2.63–2.66 times as long as wide; apical field about 0.6 times as long as total wing length, with 2 pigmented oblique linear sclerites; radial cell well developed, elongate, length 4.1 times as long as wide, with inner posterobasal angle acute; cross-vein r3 long, length about 2.2 times length of radial cell, horizontal and arising away from r4, which is slightly straight and complete; base of RP very long, extending to wing base; R-M loop forming narrowly acute angle; medial spur arise and then straight; medial field with five free veins; MP3+4 branching in 2 long veins; wedge cell length about 2.8 times its width (Fig. 9).

Tarsomeres 1–3 elongate, tarsomere 4 smaller than precedents; pretarsal claws simple; empodium short, not extending between claws; tarsomeres 2, 3 and 4 lobate (Fig. 18).

**Figure 17–19.**
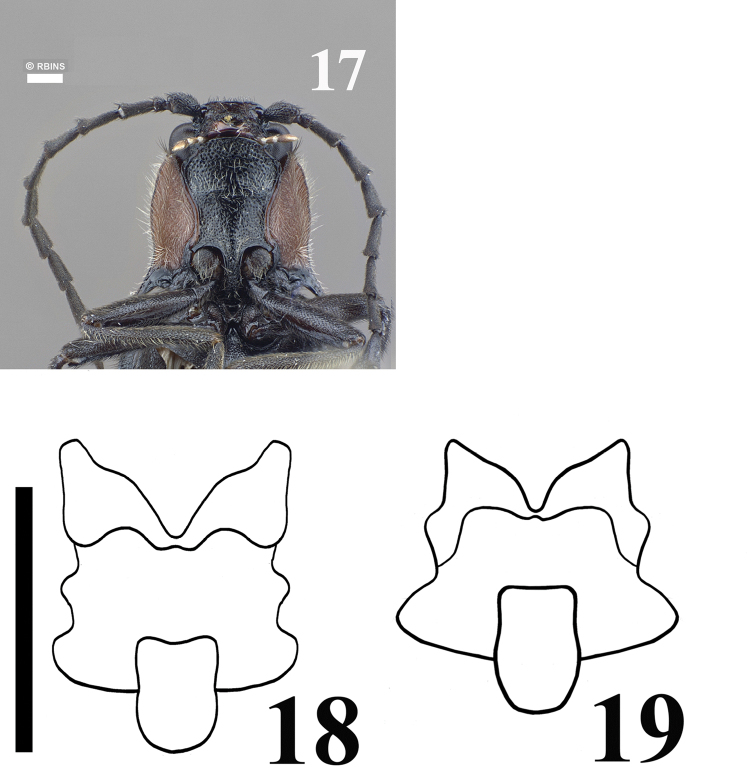
17 Ventral view of *Malalcahuello ocaresi*. **18–19** SEM illustration of tarsomeres of: *Malalcahuello ocaresi* (**18**), *Campyloxenus pyrothorax* (**19**). Scale bar = 0.5 mm.

Female genitalia: bursa copulatrix globular, space shuttle shape from above, one spherical thicker gold gland medially, sides of bursa with 2–3 spinules semi-curved (Figs 11, 12, 13).

Male genitalia: aedeagus symmetrical, phallobase broadly rounded; each paramere with a lateral hook at apex; median lobe attached to parameres both dorsally and ventrally (Fig. 22).

**Figures 20–23.**
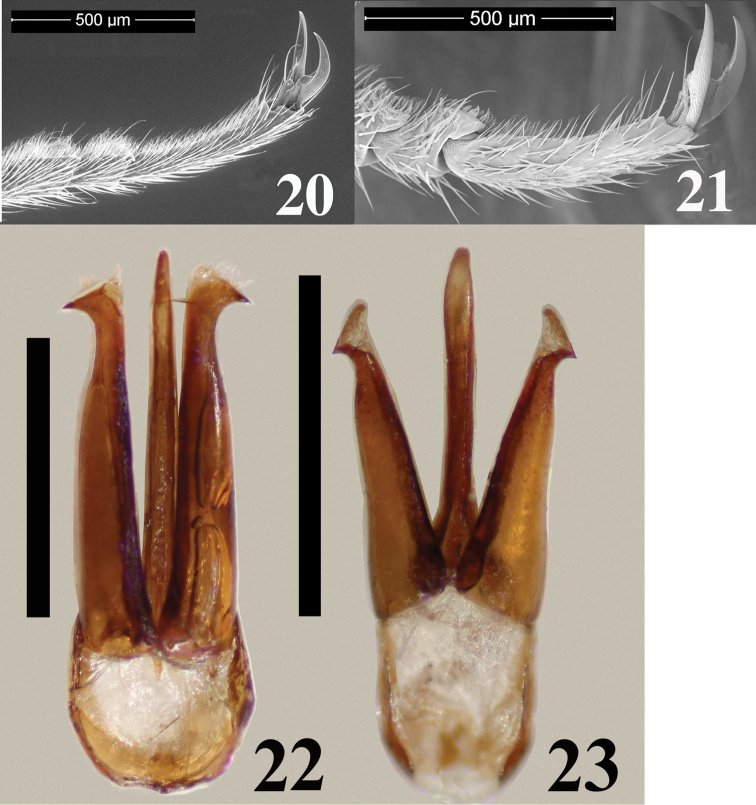
**20–21** Tarsomeres of *Malalcahuello ocaresi* (**20**), *Campyloxenus pyrothorax* (**21**) **22–23** Male genitalia of *Malalcahuello ocaresi* (**22**), *Campyloxenus pyrothorax* (**23**). Scale bar = 0.5 mm.

#### Distribution.

Southern Chile: provinces Ñuble and Malleco.

### Malalcahuello ocaresi sp. n.

Taxon classificationAnimaliaColeopteraElateridae

http://zoobank.org/B58F21AD-7022-415E-AA42-521FF0F97973

Figs 1 , 2 , 5 , 7 , 9 , 11–13 , 17 , 18 , 20 , 22

#### Etymology.

This species is named in honor of Sergio Ocares Figueroa, long time friend, and excellent insect collector from Los Lleuques, Region VIII, Chile.

#### Description.

Holotype: male, total body length 11.63 mm, width 3.00 mm. (Fig. 1).

Head and mandibles dark brown; antenna same color as head.

Pronotum about 0.76–0.99 times as long at midline as its greatest width; reddish, with long, gold semi-decumbent curved hairs; punctate, punctures separated for more than one own diameter; prosternal spine about 0.63 times as long as diameter of procoxal cavity.

Scutellum black or same color than elytra, anterior border sometimes darker; with long, thin, gold semi-decumbent hairs; Elytra about 2.54–2.81 times as long at midline as its greatest width; black or dark brown; elytral punctures closely aligned in rows forming a defined striae; posterior edge of mesosternal cavity excavate. Legs brown, vestiture black; tarsomeres 2, 3 and 4 with lobes, lobes of tarsomeres 3 and 4 spongiose and extending well beyond tarsomere length apically (Fig. 18).

Aedeagus. Length 1.89 mm, and 0.35 mm wide; parameres apex globose with a hook, with at least 3 strong setae (Fig. 22).

#### Distribution.

Southern Chile. Provinces: Ñuble and Malleco.

#### Remarks.

Variation within species ranges in males 11.06-13.09 mm in length, and females 15.25-15.75mm in length; elytra color black to dark brown.

#### Type material.

HOLOTYPE: ♂Chile VIII Region/ Las Trancas 18/24/xii/2005 Malaise Trap/ Arias & Ocares UC Berkeley 36°54'26"S, 71°29'36"W //EMEC117539// [MNNC]

PARATYPES:♀ Chile Malleco/ Tolhuaca Curac./ 15.i.1950 L. Peña// Alotipo (red) 38°18'36"S, 71°38'42"W // EMEC113596// [SRC]

♀Chile Malleco/ Rio Blanco Cur. / 15.ii.1954 L. Peña E. // 38°13'00"S, 72°20'00"W // EMEC10006010// [JEB]

♀Chile Malleco/ Río Blanco Termas / 15.i.1974 L. Peña // 38°13'00"S, 72°20'00"W //Paratipo// (wings on a card) // Ex-COLECCION / Jorge Valencia / JVCC / Chile 003256 // EMEC10006013// [JEB]

♀Chile Malleco /I 1996/ A. /Ugarte // 38°13'00"S, 72°20'00W" //EMEC10006017// [MNHN]

♂CHILE Malleco Pr.:/ Malalcahuello, 13.7 km E/ of on road to Lonquimay./ 1565m 38°26.15'S/ 71°29.26'W// 24.xii.1996–6.ii.1997. /Nothofagus pumilio/Araucaria araucana/ forests w/Chusquea // FMHD #96-234. Flight intercept trap/ A. Newton/ & M. Thayer 1978// EMEC117552 //[MNNC]

♂Chile VIII Region/ Las Trancas 18/24/xii/2005 Malaise Trap/ Arias & Ocares UC Berkeley 36°54'26"S, 71°29'36"W //EMEC10006015// [RBINS]

♂Chile VIII Region/ Las Trancas 18/24/xii/2005 Malaise Trap/ Arias & Ocares UC Berkeley 36°54'26"S, 71°29'36"W //EMEC113597// [ETA]

♂Chile Ñuble Shangrila/ 6-11/XII.1998. / J Mondaca / 36°54'26"S, 71°29'36"W //EMEC109681// [EMEC]

♂CHILE Malleco Pr.:/ Malalcahuello, 13.7 km E/ of on road to Lonquimay./ 1565m 38°26.15'S/ 71°29.26'W// 24.xii.1996–6.ii.1997. /Nothofagus pumilio/Araucaria araucana/ forests w/Chusquea // FMHD #96-234. Flight intercept trap/ A. Newton/ & M. Thayer 1978// EMEC110090 //[FMNH]

♂Las Trancas / VIII Region Chile. Enero 1982/ Coll. T. Curkovic. // 36°54'26"S, 71°29'36"W // EMEC10006011// [BMNH]

♂Chile Ñuble Prov. / Las Trancas 19.5 km/ E Recinto. 1250, / trap site 647/ 10.xii.82–3.i.1983/ Nothofagus Forests/ A. Newton & M. Thayer // Window/trap 647 36°54'26"S, 71°29'36" //EMEC10006012 //[FMNH]

♂Chile Malleco/ Rio Blanco Cur./ 15.ii.1954 L. Peña E. [JEBT] Ex-COLECCION / Jorge Valencia / JVCC / Chile 001619 // COLECCION JEBC / Juan Enrique / Barriga-Tuñon / Chile 0203579 // 38°13'00"S, 72°20'00" //EMEC10006013// [JEB]

♂Chile 1400 mts/ prov. Malleco/ vn. Lonquimay/ 22-Dic-1994/ Leg. J. E. Barriga// Coleccion JE Barriga/ // 38°22'36"S, 71°35'00"// 46023//EMEC10006014// [ANIC]

♂CHILE REGION IX (LA ARAUCANIA)/ P.N. Villarrica. Paso Mamuil Malal/ Araucaria Picnic area/ S39°34 283, W71°29 908, 1100 m/ 19.January.2006 sweeping & beating/ A.B.T. Smith, M. J.Paulsen // in a vial// EMEC10006016// [ETA].

Other Material studied: *Campyloxenus pyrothorax* Fairmaire et Germain, 1860.

♂Chile Bío Bío / Los Angeles/ 26-12-1940 /37°28'S 72°21'W; CHILE Region IX/ Parque Huerquehue, 2825'39°92'S/71°43.323'W, xii-11-2001. Malaise trap / Arias et al Berkeley; Chile Bío Bío/ Los Angeles/ 26-12-1940/ B Orellana Colector 37°28'S 72°21'W; CHILE Region IX/ Parque Huerquehue, 2825'39°92'S/71°43.323'W xii-11-2001. Malaise trap / Arias et al., Berkeley; Chile VIII Region/ Las Trancas 18/24/xii/2005 Arias & Ocares UC Berkeley 36°54'26"S, 71°29'36"W; 52.- Chile X Region/ Oncol Park/ Calfuco Way/ Fogging 14:13PM. 150cc / l/ 07 / I / 2007. 515m 14°C / 39°42.114/ 73°19.244/ *Saxegothaea conspicua* 35m/ Arias et al., UCB; 8-CHILE VIII Region/ PN Nahuelbuta. Pichinahuel Exit/ 37°48.341'S/ 73°02.112'W /1215m/ 05.XII.2001. Canopy Fogging GT/ *Araucaria araucana* F/ Arias & Andrews et al., UCB; ♀ (2): CHILE REGION IX (LA ARAUCANIA)/ P.N. Villarrica. Paso Mamuil Malal/ Araucaria Picnic area/ S39°34'283"W 71°29'908", 1100 m/ 19 January 2006 sweeping & beating/ A.B.T. Smith, M.J. Paulsen.

### Key to separate Chilean genera of Campyloxeninae

**Table:** 

1	Clypeus more than 4 times as long as its width (Fig. 7); pronotum with long, decumbent vestiture, lacking luminous spots and a deep circular impression basally (Figs 1, 2); lobe of tarsomere 4 extending apically about 0.7 times length of tarsomere 4 (Fig. 20)	***Malalcahuello* gen. n.**
–	Clypeus less than 4 times as long as its width (Fig. 8); pronotum with short, erect vestiture, with luminous spots and with a deep circular impression basally (Figs 3, 4); lobe of tarsomere 4 extending apically less than 0.5 times length of tarsomere 4 (Fig. 21)	***Campyloxenus* Fairmaire & Germain, 1860**

## Discussion

*Campyloxenus* and *Malalcahuello* belong to the subfamily Campyloxeninae since they share the following characters (additionally to those of Costa 1975): body somewhat soft; long serrate antennae from antennomere 3; stout and protruding posterior angles; anterior region of mesosternum anteriorly produced and bilobate (arrow in Fig. 7), anterior articulating surfaces of mesosternum well-developed (arrow in Fig. 8); mesosternal cavity oval, not deep, open to mesepimerum and to mesepisternum; pre-scutum v shape, scutellum not notched and somewhat subrectangular (Figs 18, 19); tarsomeres 1–4 with lobes, tarsomeres 1–2 with very small lobes (Figs 20, 21); sexual dimorphisms, females are larger than males and present shorter antennae. Both *Campyloxenus* and *Malalcahuello* are monotypic genera.

*Malalcahuello* differs from *Campyloxenus* by the following (contrasting characters for *Campyloxenus* in parentheses): frontoclypeal carina frontally rugulose (frontoclypeal carina frontally not rugulose); lacking bioluminescent organs (exhibits bioluminescent organs); clypeus about 4.8 times as long as wide (clypeus about 3.6 times as long as wide); clypeus and labrum with thin hairs (clypeus and labrum with thick hairs); pronotal sides slightly sinuated (Figs 1, 2) (pronotal sides strongly sinuated (Figs 3, 4)); lobe of tarsomere 4 extending apically about 0.7 times length of tarsomere 4 (lobe of tarsomere 4 extending apically less than 0.4 times length of tarsomere 4); bursa copulatrix shuttle space-shape (Figs 11–13) (bursa copulatrix elongate (Figs 14–16)).

Members of the Elateridae generally exhibit a hard body, but members of the subfamily Campyloxeninae exhibit a soft-body trait that is also found within the Elateriformia, in Dascillidae, Elmidae, Ptilodactylidae and Psephenidae (Bocakova et al. 2007). Within the subfamily Campyloxeninae, only *Campyloxenus* exhibits bioluminescent organs. These are lacking in *Malalcahuello*. Bioluminescence is limited to the tribes Pyrophorinae and Hapsodrilini within the Elateridae (Colepicolo-Neto et al. 1986), and the genus *Balgus* (Costa, 1984) that has been placed in Thylacosterninae (Vahtera et al. 2009). Most species of Coleoptera possessing bioluminescent organs exhibit soft bodies are members of the cantharoid section of Elateriformia. Kundrata et al. (2014) indicated multiple origins for the soft-bodied trait and bioluminescent organs. *Malalcahuello* lacks bioluminescent organs and its body is harder than *Campyloxenus*. Future molecular studies of endemic Campyloxeninae may elucidate their systematic position within the Elateriformia.

## Supplementary Material

XML Treatment for Malalcahuello

XML Treatment for Malalcahuello ocaresi
